# Dissecting the genetic heterogeneity of myopia susceptibility in an Ashkenazi Jewish population using ordered subset analysis

**Published:** 2011-06-17

**Authors:** Claire L. Simpson, Robert Wojciechowski, Grace Ibay, Dwight Stambolian, Joan E. Bailey-Wilson

**Affiliations:** 1 Inherited Disease Research Branch, National Human Genome Research Institute, National Institutes of Health, Baltimore, MD; 2 Department of Medicine, Johns Hopkins School of Medicine, Baltimore, MD; 3 Department of Epidemiology, Johns Hopkins Bloomberg School of Public Health, Baltimore, MD; 4 Department of Ophthalmology, University of Pennsylvania, Philadelphia, PA

## Abstract

**Purpose:**

Despite many years of research, most of the genetic factors contributing to myopia development remain unknown. Genetic studies have pointed to a strong inherited component, but although many candidate regions have been implicated, few genes have been positively identified.

**Methods:**

We have previously reported 2 genomewide linkage scans in a population of 63 highly aggregated Ashkenazi Jewish families that identified a locus on chromosome 22. Here we used ordered subset analysis (OSA), conditioned on non-parametric linkage to chromosome 22 to detect other chromosomal regions which had evidence of linkage to myopia in subsets of the families, but not the overall sample.

**Results:**

Strong evidence of linkage to a 19-cM linkage interval with a peak OSA nonparametric allele-sharing logarithm-of-odds (LOD) score of 3.14 on 20p12-q11.1 (ΔLOD=2.39, empirical p=0.029) was identified in a subset of 20 families that also exhibited strong evidence of linkage to chromosome 22. One other locus also presented with suggestive LOD scores >2.0 on chromosome 11p14-q14 and one locus on chromosome 6q22-q24 had an OSA LOD score=1.76 (ΔLOD=1.65, empirical p=0.02).

**Conclusions:**

The chromosome 6 and 20 loci are entirely novel and appear linked in a subset of families whose myopia is known to be linked to chromosome 22. The chromosome 11 locus overlaps with the known Myopia-7 (MYP7, OMIM 609256) locus. Using ordered subset analysis allows us to find additional loci linked to myopia in subsets of families, and underlines the complex genetic heterogeneity of myopia even in highly aggregated families and genetically isolated populations such as the Ashkenazi Jews.

## Introduction

Myopia is a leading cause of visual impairment worldwide, affecting approximately 1 in 4 US adults [1-3]. Myopia is much more common in some other populations, especially in East Asian cities where its prevalence has reached epidemic proportions of 80% or more in young adults [4-7]. Although much of this increase has been attributed to environmental factors [8], genetic epidemiological studies have firmly established that myopia (and refractive errors in general) also have strong heritable components [9-20]. Genetic and environmental factors both clearly influence myopia development, and it is possible that interactions between genes and the environment may help explain the high heritability and the recent rapid changes in prevalence [21,22].

Many genetic linkage studies of myopia, predominantly focusing on highly penetrant, severe manifestations in highly ascertained families have identified several regions across the genome that were linked to myopia including 2q37.1 [23], 4q22-q27 [24], 5p15.3-p15.2 [25], 10q21 [26], 18p11.31 [27], 12q21-q23 [28], 7q36 [29], and 17q21-q22 [30]. The more common, less severe form of myopia, has also been the subject of several linkage analyses that have found significant linked regions on 22q12 [31,32] and 2q37 [33,34]. There have also been several studies of ocular refraction, defined as a quantitative trait, in families. Significant linkage to ocular refraction has been reported on 3q26, 4q12, 8p23 and 11p13 [34,35], 1p36 [36-38] and 7p15 [39]. Suggestive evidence of linkage of ocular refraction to 22q in the Beaver Dam Eye Study [40] has supported the significant linkage observed for myopia to this region. Similarly, suggestive evidence of linkage of myopia to 3q26 and 8p23 [41] in an Amish data set and to 11p13 in a Caucasian-American data set [42] have supported the significant linkage of ocular refraction seen in British Twins [35] to these regions. These results underline the large amount of genetic heterogeneity which is well recognized in the field [29].

We have previously reported genomewide linkage studies of ocular refraction phenotypes performed in Ashkenazi Jewish families [31,32,36]. To investigate whether there was substantial genetic heterogeneity of myopia in these families, we used ordered subset analysis [43] in an attempt to find additional linked regions in these families which had previously not been detectable in standard linkage analysis.

## Methods

### Families

Family recruitment and selection criteria have been reported elsewhere and are summarized here [31]. In brief, participants were recruited into the Myopia Family Study primarily from the Lakewood, NJ area. All participating individuals were of Orthodox Ashkenazi Jewish cultural/religious heritage (individuals of Sephardic Jewish origin and their offspring were not included in the study). To be eligible for the study, a nuclear family had to contain only one myopic parent and at least one myopic offspring. These criteria were established to enhance selection of autosomal-dominantly transmitted myopia within families. Larger pedigrees were then formed by extending nuclear families through first- and second-degree relatives. Extended families were then selected for the linkage study if a) at least one affected pair of relatives besides a single parent-offspring pair existed and 2) biologic specimens were available for at least these affected individuals.

### Phenotyping

Sixty-three multiplex Ashkenazi Jewish families were included in the study. Eligibility for family participation in the study required an index case whose spherical equivalent refraction was −1.00 Diopters (D) or lower in both eyes (as long as there was −1.00 D or lower in each meridian if astigmatism was present) and had no history of a systemic or ocular disease that might predispose to myopia, including premature birth. Cycloplegic refractions were used for index cases under 50 years of age while manifest refractions were used for those above age 50. The same classification scheme was used to determine affection status for all individuals in the pedigrees, and subjects who did not meet this standard were regarded as unaffected. If a subject was reported to have been myopic but this diagnosis could not be confirmed with either medical records, measurement of the prescription of a pair of eyeglasses, or current physical examination, the individual was treated as being of “unknown” phenotype.

Because of the normal developmental changes in refraction during childhood and the potential for misclassification, a more stringent approach to classification of affected versus unaffected subjects was used for the groups of individuals aged 6–10 years and 11–20 years. All individuals with a −1.00 D or lower spherical equivalent were considered affected, as above, regardless of age. However, subjects in the group of individuals aged 6–10 years with a +2.00 spherical equivalent refraction or higher in both eyes were classified as unaffected, since they are not likely to develop myopia. Individuals in this age group with a spherical equivalent between +2.00 and −1.00 were classified as “unknown.” Individuals in the group of subjects aged 11–20 years with +1.50 spherical equivalent or higher in both eyes were classified as unaffected. Any individual with a spherical equivalent of between +1.50 and −1.00 in this age group was placed in the “unknown” class. This conservative approach balances the power loss that results from our lack of a good segregation-analysis model of age-dependent penetrance and the concomitant confusion about appropriate genotype probabilities for young unaffected subjects, with the power loss resulting from the classification of normal children as “unknown.”

### Microsatellite genotyping

High–molecular-weight DNA was isolated from buffy coats with a kit (Puregene; Qiagen Inc., Valencia, CA). Samples were stored in a DNA repository under a unique code. Altogether 481 DNA samples from 63 families including 220 affected males and 141 affected females were genotyped at the Center for Inherited Disease Research (CIDR; Johns Hopkins University, Baltimore, MD). The first 44 families were genotyped by CIDR using automated fluorescent microsatellite analysis. PCR products were sized on an ABI 3700 sequencer (Life Technologies Inc, Carlsbad, CA). The marker set used was a modification of the Cooperative Human Linkage Center marker set, version 9 (387 markers; average spacing 9 cM; average heterozygosity 0.76). The final 19 families were genotyped at a later date also by CIDR using 402 markers from the modified Cooperative Human Linkage Center version 9. All genotyping was performed blind to clinical status.

Since the two genome-wide linkage scans were performed at different times, we felt it was problematic to attempt to combine them by reconciling the genotypes at the same loci in the two data sets. Instead, for each microsatellite marker, we created two dummy markers with a genetic distance of 0 between them. The first data set had real genotypes at dummy marker 1 and missing data at dummy marker 2. Individuals in the second data set were coded with missing data at dummy marker 1 and their real genotypes at dummy marker 2.

### Linkage analyses

These analyses have been described elsewhere and are summarized here [31,32]. Multipoint non-parametric linkage analyses were performed with GENEHUNTER-PLUS software [44,45] to obtain family-specific non-parametric linkage scores (NPL scores).

### Ordered subset analysis

To address genetic heterogeneity we used linkage to 22q12 as a covariate and performed Ordered Subset Analysis (OSA) [43]. Non-parametric linkage methods are powerful to detect loci that contribute to risk in a large proportion of families, but less powerful when the proportion of linked families is small. By conditioning on our identified locus, we can account for genetic heterogeneity across families and increase power to detect linkage to other loci. Multipoint NPL scores were used to take advantage of extended pedigree structure, ranking families by maximum non-parametric linkage to 22q12. The method ranks families based on their NPL score -first in ascending order then in descending order -to find an appropriate subset that maximizes evidence of linkage [43]. The ‘optimal-slice’ yields the maximum logarithm-of-odds (LOD) score determined by a subset of any size of adjacent families based on their covariate distribution (not necessarily including endpoints), allowing exclusion of families with extreme NPL scores. Interpretation of OSA LOD scores is not straightforward since the OSA LOD is dependent on the overall evidence for linkage in the complete sample. OSA LOD scores are not equivalent to traditional parametric linkage LOD scores. To evaluate the significance of the OSA LOD scores in this study, we used the method developed by Hauser et al. [43] of examining the difference in the overall and conditional OSA LOD scores, which is similar to the method of Cox et al. [46]. Empirical p values were calculated to assess OSA LOD scores by performing OSA analyses on 10,000 randomizations of family order. This permutation test is significant when the covariate-defined subset yields stronger evidence of linkage than observed in the randomly assigned family subsets and indicates how likely it is to obtain by chance a subset-based OSA LOD score greater than or equal to the observed OSA maximum LOD score. Using a Bonferroni correction for multiple testing to account for the two models used (ascending and descending NPL-scores) [47], gives an adjusted significance threshold of 0.025 for assessing the statistical significance of the change in the OSA LOD score from the unconditional test (ΔLOD) . If there is little evidence for linkage in the overall sample, the empirical p value can still be quite significant even when there is only moderate evidence for linkage in the subset. Conversely, if there is strong but widely dispersed evidence for linkage, then the empirical p values may not achieve statistical significance. Results must therefore be considered in the context of the evidence for linkage in the entire data set.

## Results

**Figure 1:**
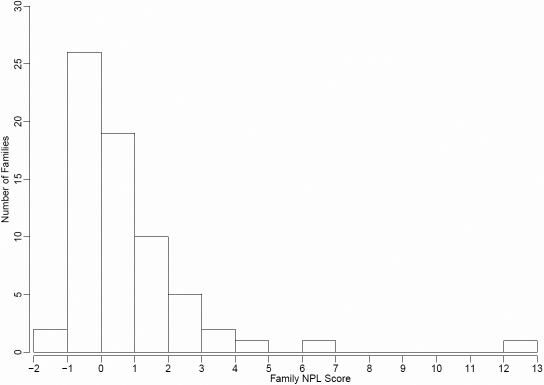
Distribution of individual family NPL scores in the Ashkenazi Jewish families.

**Figure 2:**
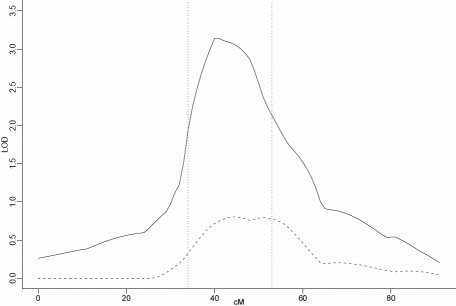
Nonparametric LOD score plot for chromosome 20. LOD scores for the overall sample (n=63 [dashed line]) and the subset with maximum NPL score between 2.25 and 0.6 (n=20 [solid line]) are presented. The 1-LOD-unit down support interval (34–53 cM) is marked by the dotted vertical line.

Individual family NPL scores calculated by GENEHUNTER-PLUS for chromosome 22 at the position of the known linkage peak were used as the covariate in the non-parametric OSA. The distribution of individual family NPL scores can be seen in Figure 1. There were 565 affected individuals, 355 males and 210 females (male:female ratio=1.69:1), and the mean number of affected individuals per family was 8.43. Nominally significant increases in OSA LOD score (compared to the 10,000 permutation results) were observed in three regions of the genome, on chromosomes 6q22-q24, 11p14-q14 and 20p12-p11. When ordering families by descending maximum non-parametric linkage to chromosome 22, a maximum OSA LOD score of 3.14 was obtained at D20S470 (42.27cM) in the 20 families with family-specific NPL scores on 22q between 2.25 and 0.6. This was an increase of 2.139 in the OSA LOD compared with the unconditional test using all families and this increase was nominally significant by permutation testing (p=0.029). The 1-LOD-unit support interval for this peak is 19 cM wide, from 34 to 53 cM (Figure 2 and Table 1). However, this peak was not quite significant (Bonferroni-corrected significance threshold=0.025) after correction for multiple tests. This subset of families contained 90 affected males and 62 affected females (male:female ratio=1.45:1). Clinical characteristics of this subset are detailed in Table 2.

**Table 1:** Maximum OSA LOD scores in the 63 families conditioning on linkage to chromosome 22.

**Region**	**Slice**	**cM**	**Max OSA LOD**	**NPL**	**ΔLOD**	**Empirical p value**	**Families**	**Proportion of families**
6q22-q24	Descending	142.57	1.74	0.09	1.65	0.015	36	0.58
11p14-q14	Descending	68.72	2.36	0.68	1.68	0.035	45	0.71
20p12-p11	Optimal (descending)	42.27	3.14	0.75	2.39	0.029	20	0.32

**Table 2:** Clinical characteristics of the subsets identified by OSA.

**Family rank (Ascending by NPL)**	**NPL for linkage to Chromosome 22**	**Number of affected**	**Male:Female ratio**
**Chromosome 20 subset**			
38	0.64568	6	2:1
39	0.65514	13	1:1.17
40	0.713566	10	1.5:1
41	0.765529	5	1:1.5
42	0.891322	9	2:1
43	0.94953	7	1.33:1
44	1.1722	8	7:1
45	1.247382	8	1.67:1
46	1.304162	7	1.33:1
47	1.306068	8	1:1
48	1.334312	6	1:1
49	1.336603	5	1:1.5
50	1.412251	2	1:1
51	1.557204	10	4:1
52	1.692256	4	3:1
53	1.851772	7	2.5:1
54	2.113614	11	4.5:1
55	2.185684	7	1.33:1
56	2.219589	13	1:1.6
57	2.242624	6	1:2
**Chromosome 11 subset**			
19	−0.18266	8	1:3
20	−0.13992	12	5:1
21	0	3	2:1
22	0	5	0:5
23	0	4	0:5
24	0	3	2:1
25	0.100143	16	2.2:1
26	0.106514	8	3:1
27	0.109667	8	7:1
28	0.129182	8	3:1
29	0.15711	9	1:1.25
30	0.180609	8	3:1
31	0.205917	9	2:1
32	0.282509	9	0:3.5
33	0.351538	12	3:1
34	0.35623	5	0:5
35	0.383718	16	7:1
36	0.490716	9	2:1
37	0.504382	15	2.75:1
38	0.64568	6	2:1
39	0.65514	13	1:1.17
40	0.713566	10	1.5:1
41	0.765529	5	1:1.5
42	0.891322	9	2:1
43	0.94953	7	1.33:1
44	1.1722	8	7:1
45	1.247382	8	1.67:1
46	1.304162	7	1.33:1
47	1.306068	8	1:1
48	1.334312	6	1:1
49	1.336603	5	1:1.5
50	1.412251	2	1:1
51	1.557204	10	4:1
52	1.692256	4	3:1
53	1.851772	7	2.5:1
54	2.113614	11	4.5:1
55	2.185684	7	1.33:1
56	2.219589	13	1:1.6
57	2.242624	6	1:2
58	2.676218	7	1.33:1
59	3.07075	8	1:1
60	3.420423	10	1:1
61	4.934796	25	1.5:1
62	6.694279	9	1:2
63	12.19355	17	1:1.83
**Chromosome 6 subset**			
28	0.129182	8	3:1
29	0.15711	9	1:1.25
30	0.180609	8	3:1
31	0.205917	9	2:1
32	0.282509	9	0:3.5
33	0.351538	12	3:1
34	0.35623	5	0:5
35	0.383718	16	7:1
36	0.490716	9	2:1
37	0.504382	15	2.75:1
38	0.64568	6	2:1
39	0.65514	13	1:1.17
40	0.713566	10	1.5:1
41	0.765529	5	1:1.5
42	0.891322	9	2:1
43	0.94953	7	1.33:1
44	1.1722	8	7:1
45	1.247382	8	1.67:1
46	1.304162	7	1.33:1
47	1.306068	8	1:1
48	1.334312	6	1:1
49	1.336603	5	1:1.5
50	1.412251	2	1:1
51	1.557204	10	4:1
52	1.692256	4	3:1
53	1.851772	7	2.5:1
54	2.113614	11	4.5:1
55	2.185684	7	1.33:1
56	2.219589	13	1:1.6
57	2.242624	6	1:2
58	2.676218	7	1.33:1
59	3.07075	8	1:1
60	3.420423	10	1:1
61	4.934796	25	1.5:1
62	6.694279	9	1:2
63	12.19355	17	1:1.83

**Figure 3:**
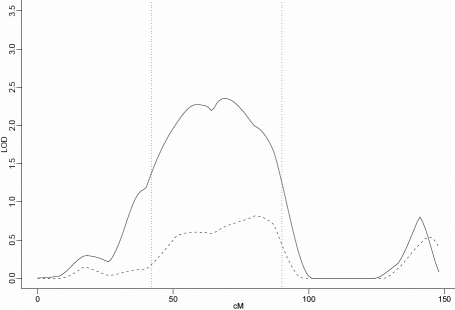
Nonparametric LOD score plot for chromosome 11. LOD scores for the overall sample (n=63 [dashed line]) and the subset with maximum NPL score between 12.2 and −0.3 (n=45 [solid line]) are presented. The 1-LOD-unit down support interval (42–90 cM) is marked by the dotted vertical line.

Also found by ordering families by descending maximum non-parametric linkage to chromosome 22 was a locus with a maximum OSA LOD score of 2.36 obtained at D11S1344 (68.72 cM) in a subset of 45 families with the strongest evidence of nonparametric linkage to chromosome 22 with NPL score ranging from 12.2 to −0.3. This was an increase of 1.68 in the OSA LOD compared with the unconditional test using all families and this increase was nominally significant by permutation testing (p=0.035). The 1-LOD-unit support interval spans 48cM, from 42 to 90 cM (Figure 3 and Table 1). This subset of families contained 197 affected males and 130 affected females (male:female ratio=1.52:1; Table 2).

**Figure 4:**
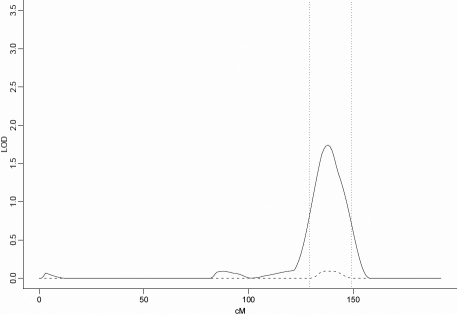
Nonparametric LOD score plot for chromosome 6. LOD scores for the overall sample (n=63 [dashed line]) and the subset with maximum NPL score between 12.2 and −0.3 (n=36 [solid line]) are presented. The 1-LOD-unit down support interval (129–149 cM) is marked by the dotted vertical line.

The third locus, at 6q22-q24, was also found by ordering families by descending maximum non-parametric linkage to chromosome 22. This locus had a maximum OSA LOD score of 1.76 at D6S1009 (142.57 cM), an increase of 1.65 in the OSA LOD compared to the unconditional test using all families and this increase was significant by permutation testing (p=0.02) after correction for multiple tests. This subset contained 36 families with the strongest evidence of nonparametric linkage to chromosome 22. The 1-LOD-unit support interval was 20 cM, from 129 to 149 cM. (Figure 4 and Table 1).This subset of families contained 254 affected males and 160 affected females (male:female ratio=1.59:1; Table 2).

## Discussion

We have identified three regions with suggestive evidence of linkage to myopia in subsets of families already linked to chromosome 22: a locus on 6q22-q24 which seems to be entirely novel; a locus on 11p14-q14 which, although large, does overlap with Myopia-7 (MYP7, OMIM 609256); and another locus on 20p12-p11.

The 20p12-p11 locus appears about one third of our Ashkenazi Jewish families. This locus overlaps with loci known to be associated with other ocular traits such as keratoconus and posterior polymorphous corneal dystrophy [48], age-related macular degeneration [49,50] and juvenile onset primary open angle glaucoma [51]. Suggestive evidence of linkage of myopia to this region was previously found in a set of African-American and White families [42]. The change in LOD score in the OSA analyses of these Ashkenazi Jewish families was not quite significant after correcting for multiple testing, and therefore may be a type I error, but the number of families in the subset was small (20 families, 32% of total) and so it is also possible that analyzing this subset may not have had sufficient power. The fact that NPL analyses of multiple data sets (Ashkenazi Jewish, African American, and White American families) all yield suggestive evidence of linkage to myopia in this region gives support to the presence of a risk locus in this region.

The locus on chromosome 11, although broad, overlaps MYP7, a locus previously reported in a population of UK twins and of particular interest as it contains the known eye gene, paired box gene 6 (*PAX6*) [10]. So far the literature on *PAX6* polymorphisms and myopia is mixed [52-58], and it may be that this signal, and the signal found by Hammond et al. [35], is coming from another gene in this region. Suggestive evidence of linkage of myopia to this region has also been observed in another, independent set of Caucasian-American families [42] but not in an Australian set of dizygotic twins [59]. Again, the increase in OSA LOD was not significant after correcting for multiple testing but it is nonetheless interesting that this large increase in linkage evidence after OSA is observed that overlaps with a region that has been significantly linked to myopia in a different population.

The chromosome 6 locus is also novel and is the only one of these three loci to withstand correction for multiple testing. The region contains few genes, but D6S1009 is within 100 kb of the peroxisome biogenesis factor 7 (*PEX7*) gene, mutations in which can cause ocular phenotypes as part of the severe systemic syndromes Refsum disease [60,61] and rhizomelic chondrodysplasia punctata [60,62]. However, the severity and systemic nature of these disorders make severe mutations in this gene unlikely candidates for a relatively mild trait such as myopia, but it is possible that mutations with only mild effects on gene function could be involved. No other genes in the region seem like obvious candidates, but there are a few genes of unknown function and these may yet have some undiscovered biologic relevance.

All three loci were found in subsets which already had evidence of linkage to chromosome 22. There are several possible reasons why these loci are not observed in the original linkage analyses. One explanation might be that the effect on risk due to these loci is smaller than the effect on risk of the chromosome 22 locus, and thus there is not adequate power to detect these linkage signals in the complete data set, even using heterogeneity LOD scores. Another interpretation is that if there is a true statistical interaction on risk between these loci and the chromosome 22 locus, then individuals in non-chromosome 22 linked families might share alleles identical-by-descent at these novel loci without having similar phenotypes. It does appear that multiple loci may be acting together to account for the high risk of myopia in these families. It is clear from the original linkage analyses that the main effect is due to the chromosome 22 locus because the additional signals appear in families linked to this locus. Our interpretation of the results is that if these additional loci are truly affecting risk of myopia, then they may be playing a modifying role on the complex development of the eye, perhaps through multiple different regulatory mechanisms. Certainly the loci we have identified here did not have a strong enough effect to be found on their own in the original analyses. Alternatively, because these are highly selected families, it is possible that each locus has independent, non-interacting effects on risk of myopia, and risk genotypes are segregating for all of these loci in the same families because of the mode of ascertainment.

Emmetropisation is a very complex regulatory system with bio-feedback loops that are able to work at a very local level. Severing the optic nerve or blocking nerve transduction does not prevent form-deprivation myopia [63-65] and using diffusers or negative lenses to cover only half of the retina produces enlargement and myopia only on that side [66-70]. Myopia is due to a failure of these regulatory mechanisms and given the complexity of this process, which is still not well understood, there are likely to be multiple genes important to detecting, transmitting and responding to visual signals entering the eye, and to controlling grow and stop signals. This process could involve genes with large and small effects on the pathways which come together to produce myopia. In complex traits such as these, where multiple genetic loci are thought to contribute to disease risk, techniques for detecting the contributions of multiple loci are important if we are to discover the underlying genetic risk factors. OSA can only use one covariate at a time, which may be a limiting factor in traits such as myopia, where environmental factors are expected to play a significant role. Education has long been considered influential in myopia development. In this population, large differences in education of males and females exist which could lead to differential misclassification of affection status and a corresponding reduction in statistical power to detect linkage. The power of OSA depends heavily on the degree of correlation between the evidence of linkage and the levels of the OSA covariate. In studies where an environmental covariate is used such as age-at-onset, the mean value of all affected family members is typically used as the covariate value in the analysis. Power, therefore is dependent on the extent to which phenotypic variability between families reflects underlying genetic heterogeneity. In our analysis, our covariate was itself a linkage signal, and so the extent to which this varies between families will be closely correlated with the overall genetic heterogeneity in the sample.

Association testing of the subsets under the identified peaks found only nominal evidence of association which was not robust to correction for multiple testing. However, since this is a panel of microsatellites designed for linkage, there is not sufficient density of markers to have any power for association. To further investigate these loci, either fine-mapping of the linked regions using a dense panel of single nucleotide polymorphisms (SNPs) or targeted sequencing of the region in selected individuals would be useful approaches to try and narrow down the variants responsible for the signal. Given the current advances in sequencing technology, whole genome sequencing of appropriate individuals from these families may be more cost effective than targeted sequencing or custom genotyping.

Using ordered subset analysis allowed us to find additional loci linked to myopia in subsets of Ashkenazi Jewish families, and underlines the complex genetic heterogeneity of myopia even in highly aggregated families and genetically isolated populations. It is also of note that when these data were analyzed as the refractive error quantitative trait, linkage to the 22q12 locus was not significant and instead a locus on 1p36 was strongly significant [36,37]. This emphasizes that data sets such as these provide rich opportunities for the further elucidation of genetic risk factors in myopia and refractive error.
